# Supplementary material for: Agents of Campylobacteriosis in Different Meat Matrices in Brazil
Source: Int J Environ Res Public Health. 2022 May 17;19(10):6087. doi: 10.3390/ijerph19106087 (PMC9140573; doi:10.3390/ijerph19106087)
Supplement: Supplementary file 1 [file ijerph-19-06087-s001.zip › ijerph-1670566-supplementary.pdf]

**Table S1.** Virulence profiles of 81 *Campylobacter* strains isolated from meat matrices in Brazil.

| Virulence Profiles | Source | Genes | Strains | | | TOTAL |
|---|---|---|---|---|---|---|
| | | | *C. jejuni* | *C. coli* | *Campylobacter* spp. | |
| V1 | BL, PS | *cadF* | - | $1^{PS}$ | $1^{BL}$ | 2 |
| V2 | BL, PS, CL, MM | *luxS* | $1^{BL}$ | $1^{BL}$ | $1^{BL}$, $1^{PS}$, $1^{CL}$, $1^{MM}$ | 6 |
| V3 | BL, MM | *dnaJ* | - | - | $1^{BL}$, $1^{MM}$ | 2 |
| V4 | BL | *cadF, dnaJ* | - | 1 | - | 1 |
| V5 | CL | *flaA, cadF* | - | - | 1 | 1 |
| V6 | MM | *ciaB, luxS* | - | - | 1 | 1 |
| V7 | MM | *luxS, dnaJ* | - | - | 1 | 1 |
| V8 | BL, PS | *pldA, cadF, luxS* | - | - | $1^{BL}$, $3^{PS}$ | 4 |
| V9 | BL | *pldA, cadF, dnaJ* | - | - | 1 | 1 |
| V10 | BL | *flaA, cadF, luxS* | - | - | 1 | 1 |
| V11 | PS | *ciaB, cadF, luxS* | 1 | - | - | 1 |
| V12 | PS | *ciaB, luxS, dnaJ* | - | 1 | 2 | 3 |
| V13 | CL | *flaA, ciaB, luxS* | - | 1 | - | 1 |
| V14 | CL | *flaA, cadF, dnaJ* | - | 1 | - | 1 |
| V15 | CC | *ciaB, pldA, cadF* | 2 | - | 2 | 4 |
| V16 | CC | *pldA, flaA, cadF* | - | - | 1 | 1 |
| V17 | CC | *flaA, cdtA, cdtB* | 1 | - | - | 1 |
| V18 | CC | *flaA, luxS, dnaJ* | - | - | 1 | 1 |
| V19 | CC | *ciaB, pldA, flaA, cadF* | 1 | 3 | 1 | 5 |
| V20 | CC | *ciaB, pldA, cadF, dnaJ* | 1 | - | - | 1 |
| V21 | CC | *pldA, flaA, cadF, dnaJ* | - | 2 | - | 2 |
| V22 | CC | *pldA, cadF, ciaB, dnaJ* | - | - | 1 | 1 |
| V23 | CC | *flaA, cadF, cdtC, dnaJ* | - | 1 | - | 1 |
| V24 | MM | *cdtA, cdtB, cdtC, dnaJ* | - | - | 1 | 1 |
| V25 | CL | *pldA, cdtA, cdtB, luxS* | 1 | - | - | 1 |
| V26 | PS | *ciaB, cadF, luxS, dnaJ* | - | 1 | - | 1 |
| V27 | CC, PS | *ciaB, pldA, flaA, cadF, dnaJ* | - | $2^{CC}$, $1^{PS}$ | - | 3 |
| V28 | CC | *ciaB, pldA, cadF, luxS, dnaJ* | 1 | - | - | 1 |
| V29 | CC | *ciaB, cadF, cdtA, cdtB, cdtC* | 1 | - | - | 1 |
| V30 | CC | *flaA, cadF, cdtA, cdtB, cdtC* | - | 1 | - | 1 |
| V31 | BL, PS | *ciaB, pldA, flaA, cadF, luxS* | $1^{BL}$, $1^{PS}$ | $1^{PS}$ | $1^{BL}$ | 4 |
| V32 | CC | *ciaB, pldA, flaA, cadF, luxS, dnaJ* | 2 | - | - | 2 |
| V33 | CC | *ciaB, pldA, flaA, cadF, cdtC, dnaJ* | 1 | - | - | 1 |
| V34 | CC | *ciaB, pldA, flaA, cadF, dnaJ, sodB* | 1 | - | - | 1 |
| V35 | CC | *pldA, cadF, cdtA, cdtB, cdtC, dnaJ* | - | - | 1 | 1 |
| V36 | PS | *ciaB, cdtA, cdtB, cdtC, luxS, dnaJ* | 1 | - | - | 1 |
| V37 | CC | *ciaB, pldA, flaA, cadF, cdtA, cdtB, cdtC* | - | - | 1 | 1 |
| V38 | CC | *ciaB, pldA, flaA, cadF, cdtA, cdtB, dnaJ* | - | 1 | - | 1 |

| | | | | | | |
|---|---|---|---|---|---|---|
| V39 | CC | *ciaB, pldA, flaA, cadF, cdtA, luxS, dnaJ* | 1 | - | - | 1 |
| V40 | CC | *ciaB, pldA, flaA, cadF, cdtB, cdtC, luxS* | 1 | - | - | 1 |
| V41 | CC | *ciaB, flaA, cadF, cdtA, cdtB, cdtC, dnaJ* | - | 1 | - | 1 |
| V42 | CC | *ciaB, pldA, flaA, cadF, cdtA, cdtB, cdtC, dnaJ* | 2 | - | 2 | 4 |
| V43 | CC | *ciaB, pldA, flaA, cadF, cdtA, cdtB, luxS, dnaJ* | 1 | - | - | 1 |
| V44 | CC | *ciaB, pldA, flaA, cadF, cdtA, cdtB, cdtC, luxS* | 1 | - | - | 1 |
| V45 | CC | *ciaB, pldA, flaA, cadF, cdtB, cdtC, luxS, dnaJ* | 1 | - | - | 1 |
| V46 | CC | *ciaB, pldA, cadF, cdtA, cdtB, cdtC, luxS, dnaJ* | 1 | - | - | 1 |
| V47 | CC | *ciaB, flaA, cadF, cdtA, cdtB, cdtC, luxS, dnaJ* | 2 | - | - | 2 |
| V48 | CC | *pldA, flaA, cadF, cdtA, cdtB, cdtC, luxS, dnaJ* | 1 | - | - | 1 |
| V49 | CC | *ciaB, pldA, flaA, cadF, cdtA, cdtB, cdtC, luxS, dnaJ* | 2 | - | - | 2 |
| V50 | CC | *ciaB, pldA, flaA, cadF, cdtA, cdtB, cdtC, dnaJ, sodB* | 1 | - | - | 1 |
| | | **TOTAL** | **31** | **20** | **30** | **81** |

BL: bovine liver; CC: chicken carcass; CL: chicken liver; MM: minced meat; PS: pork shank.
